# Backward error analysis of the shift-and-invert Arnoldi algorithm

**DOI:** 10.1007/s00211-015-0759-9

**Published:** 2015-07-30

**Authors:** Christian Schröder, Leo Taslaman

**Affiliations:** 1 Institut für Mathematik, MA 4–5, Technische Universität Berlin, Berlin, Germany; 2 School of Mathematics, The University of Manchester, Manchester, M13 9PL UK

**Keywords:** 65F25, 65F50, 65G50

## Abstract

We perform a backward error analysis of the inexact shift-and-invert Arnoldi algorithm. We consider inexactness in the solution of the arising linear systems, as well as in the orthonormalization steps, and take the non-orthonormality of the computed Krylov basis into account. We show that the computed basis and Hessenberg matrix satisfy an exact shift-and-invert Krylov relation for a perturbed matrix, and we give bounds for the perturbation. We show that the shift-and-invert Arnoldi algorithm is backward stable if the condition number of the small Hessenberg matrix is not too large. This condition is then relaxed using implicit restarts. Moreover, we give notes on the Hermitian case, considering Hermitian backward errors, and finally, we use our analysis to derive a sensible breakdown condition.

## Introduction

Consider an implementation of the Arnoldi algorithm [4, 26]. Not much meaning can be given to the computed quantities if they deviate too much from the recurrence that underpins the algorithm in exact arithmetic:

$$\begin{aligned} AV_k=V_{k+1}\underline{H}_k,\quad \underline{H}_k=H(1:\!k+1,1:\!k). \end{aligned}$$

Luckily, good implementations, where in particular the orthogonalization is done with care, can be shown to be backward stable [3, 8, 10, 21] in the sense that the computed quantities $V_{k+1}$ and $\underline{H}_k$ satisfy an exact recurrence with a slightly perturbed matrix:

1 $$\begin{aligned} (A+\Delta A)V_k=V_{k+1}\underline{H}_k. \end{aligned}$$

This means that we can compute a basis of an exact Krylov subspace corresponding to a nearby matrix. Since the basis will in general not be perfectly orthonormal, so $V_{k+1}^HV_{k+1}^{}\ne I$, we use the term “Krylov recurrence” instead of “Arnoldi recurrence” when referring to recurrences like (1), as suggested in [24]. If *A* is Hermitian, then it can be shown that the computed basis spans a Krylov subspace associated with a perturbed *Hermitian* matrix $A+\Delta A$ [15]. There is a catch in this case, though: the small $(k+1)\times k$ matrix associated with this Krylov subspace is in general not the computed Hessenberg matrix.

In this paper we perform a similar backward error analysis of the shift-and-invert Arnoldi algorithm. For example, we show that an implementation of the Arnoldi algorithm applied to $A^{-1}$, yields computed matrices $V_{k+1}$ and $\underline{H}_k$ such that

$$\begin{aligned} (A+\Delta A)^{-1}V_k=V_{k+1}\underline{H}_k, \end{aligned}$$

and we give an upper bound for $\Vert \Delta A\Vert _2$. Perturbed versions of the shift-and-invert Arnoldi algorithm have been considered in the literature as a part of the theory of *inexact methods*, see [16, 19]. However, these results neglect that the orthonormalization is not performed exactly, and furthermore, assume bounds on linear system residuals that may be unattainable (more on this in Sect. 2). We consider more general linear system residuals and take the error from the orthonormalization into account. Our analysis of how the orthonormalization errors propagate into the shift-and-invert Krylov recurrence highlights the importance of *columnwise* backward error bounds for QR factorizations, and is thus of a different flavor than the corresponding analysis for standard Arnoldi, done in, for example [8].

We also use our error analysis to motivate when “breakdown” should be declared, that is, when $h_{j+1,j}$ may be considered to be “numerically zero”.

The algorithm we study can be divided into two main subproblems: solving linear systems and orthonormalizing vectors. We state our backward error results in such a way that they are independent of how these subproblems are being solved, but we also discuss relevant and commonly used approaches for solving these two tasks.

### Technical outline

We study floating point implementations of Algorithm 1, where *A* is assumed to be of size $n\times n$, $\sigma$ is the shift, *b* the starting vector, and *k* is the maximum number of steps we perform. Throughout the paper $\Vert \cdot \Vert$ refers to the 2-norm.
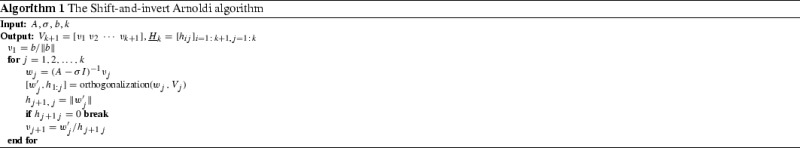


In exact arithmetic, we have

$$\begin{aligned} \text {orthogonalization}(w_j,V_j) := \left[ w_j-V_j\left( V_j^Hw_j\right) , V_j^Hw_j\right] , \end{aligned}$$

which corresponds to classical Gram–Schmidt if implemented as it stands. In floating point arithmetic, orthogonalization routines with better numerical properties, such as modified Gram–Schmidt (MGS), are usually employed.

In the *j*th iteration in Algorithm 1, a new vector $w_j$ is computed and decomposed into a linear combination of $v_1,\ldots ,v_j$ and a new component that will be the definition of $v_{j+1}$. In exact arithmetic, this can be described by the Arnoldi recurrence

$$\begin{aligned} (A-\sigma I)^{-1}v_j = V_kh_{1:\!j,j} + h_{j+1,j}v_{j+1}. \end{aligned}$$

When the corresponding thing is done in practice, however, errors are present in all steps of the computation. First, we need to solve a linear system. If we use a direct solver the matrix $A-\sigma I$ needs to be formed. We consider the rounding error in this step as part of the residual from the linear system. This does not affect the norm of the residual significantly, because the rounding error is very small,

$$\begin{aligned} \Vert \text {float}(A-\sigma I) - (A-\sigma I)\Vert <\max _{1 \le i\le n} |a_{ii}-\sigma |u\le u\Vert A-\sigma I\Vert . \end{aligned}$$

Here $\text {float}(A-\sigma I)$ refers to the computed shifted matrix and *u* is the unit roundoff. Let $r_j$ be the said residual from the linear system, so

2 $$\begin{aligned} (A-\sigma I)w_j = v_j+r_j \end{aligned}$$

is the actual linear system that has been solved. Then we have the following equality for the computed quantities:

$$\begin{aligned} (A-\sigma I)^{-1}(v_j+r_j) = w_j =V_{j+1} h_{1:\!j+1,j} + g_j, \end{aligned}$$

where $g_j$ is an error coming from the orthonormalization process. Defining

$$\begin{aligned} f_j = r_j - (A-\sigma I)g_j \end{aligned}$$

and $F_k = [f_1\; f_2\ \cdots \; f_k]$ yields a perturbed recurrence

$$\begin{aligned} (A-\sigma I)^{-1}(V_k+F_k) = V_{j+1} \underline{H}_{k}. \end{aligned}$$

We discuss the residual $r_j$ and the error $g_j$ in Sects. 2 and 3, respectively, and provide bounds for both quantities. In Sect. 4, we use these bounds in order to bound $F_k$, and subsequently the backward error for the shift-and-invert Arnoldi recurrence. In Sect. 5, we explain how the idea of implicit restarting can be used to gain further insight into the backward error. We also discuss in what sense we have Hermitian backward errors if the method is applied to a Hermitian matrix *A*. Finally, we talk about breakdown conditions: in floating point arithmetic, the test if $h_{j+1,j}=0$ in Algorithm 1 is rarely done. Instead one usually checks whether $h_{j+1,j}$ is “small enough”. This case is referred to as *breakdown*. A sensible definition of “small enough” is when the quantity is dominated by errors. We discuss this in more detail and derive backward error bounds for this case.

### Notation

The scalar $\sigma$ refers to a shift while $\sigma _{ min }(X)$ refers to the smallest singular value of *X*. The dagger notation $X^\dagger$ refers to the Moore-Penrose pseudo-inverse of *X*. The lower letter *u* is reserved to denote the unit roundoff if real arithmetic is used, and $\sqrt{5}$ times the unit roundoff if complex arithmetic is used [7]. When the matrix size is understood from the context, we denote zero matrices and identity matrices as 0 and *I*, respectively. Similarly, the vector $e_i$ denotes the *i*th column of the identity matrix whose size is understood from the context. For a matrix *X*, the lower case $x_i$ refers to the *i*th column of *X* and $X_k$ to $[x_1\; x_2\; \cdots \; x_k]$, that is, the first *k* columns of *X*.

## Errors from linear systems

In this section we discuss bounds on the residual $r_j$ from (2).

### Backward error bounds

The normwise backward error associated with a computed solution *y* of a linear system $A x = b$ is defined as

$$\begin{aligned} \eta _{A,b}(y) := \min \{\epsilon : (A+\Delta A)y=b+\Delta b, \Vert \Delta A\Vert \le \epsilon \Vert A\Vert , \Vert \Delta b\Vert \le \epsilon \Vert b\Vert \}, \end{aligned}$$

and given by the formula

3 $$\begin{aligned} \eta _{A,b}(y) = \Vert r\Vert /(\Vert A\Vert \Vert y\Vert +\Vert b\Vert ) \end{aligned}$$

where $r=Ay-b$ [20]. See also [12, p. 120]. This result is true for any vector norm $\Vert \cdot \Vert$ and its subordinate matrix norm. Thus, if we solve the linear systems in Algorithm 1, up to a backward error $\epsilon _\mathrm{bw}$, then it holds that

4 $$\begin{aligned} \Vert r_j\Vert \le (\Vert A-\sigma I\Vert \Vert w_j\Vert +\Vert v_j\Vert )\epsilon _\mathrm{bw}, \end{aligned}$$

where $r_j$ is defined in (2). If the linear systems are solved by a backward stable direct method, we have $\epsilon _\mathrm{bw}\le \phi (n)u$, where $\phi (n)$ is an algorithm dependent constant. If we are interested in the smallest possible $\epsilon _\mathrm{bw}$ such that (4) holds, then we need to compute $\Vert r_j\Vert / (\Vert A-\sigma I\Vert \Vert w_j\Vert +\Vert v_j\Vert )$. However, this may not be feasible for the 2-norm, due to the term $\Vert A-\sigma I\Vert$. In these cases we can replace $\Vert A-\sigma I\Vert$ by a lower bound (the tighter the better), and thus obtain an upper bound for $\epsilon _\mathrm{bw}$. We can for instance do a few iterations of the power method applied to $(A-\sigma I)^H(A-\sigma I)$. MATLAB’s normest function does exactly this. This would lead to a lower bound of $\Vert A-\sigma I\Vert$, since convergence is always from below. Another possibility is to use the (lower) bound in [13]. We can also bound the matrix 2-norm in terms of the corresponding infinity-norm or 1-norm. The following proposition shows that such bounds can be satisfactory for many sparse matrices, in particular those which can be permuted to banded form.

#### **Proposition 1**

Let $k_{ row }$ and $k_{ col }$ denote the maximum number of nonzero entries in a row and column of *A*, respectively. Then the following two upper and lower bounds hold:

$$\begin{aligned} \frac{1}{\sqrt{k_{ col }}}\Vert A\Vert _2&\;\le \;\Vert A\Vert _\infty \;\le \;\sqrt{k_{ row }}\Vert A\Vert _2, \\ \frac{1}{\sqrt{k_{ row }}}\Vert A\Vert _2&\;\le \;\Vert A\Vert _1\;\le \;\sqrt{k_{ col }}\Vert A\Vert _2.\\ \end{aligned}$$

#### *Proof*

We have $\Vert A\Vert _\infty = \Vert Ax\Vert _\infty$ for some *x* with $\Vert x\Vert _\infty =1$ and at most $k_{ row }$ nonzeros. We get

$$\begin{aligned} \Vert A\Vert _\infty \le \Vert Ax\Vert _\infty \le \Vert Ax\Vert _2\le \Vert A\Vert _2\Vert x\Vert _2\le \sqrt{k_{ row }}\Vert A\Vert _2, \end{aligned}$$

which is the desired upper bound for $\Vert A\Vert _\infty$. Further, we have

$$\begin{aligned} \Vert A\Vert _1=\Vert A^T\Vert _\infty \le \sqrt{k_{ col }}\Vert A^T\Vert _2=\sqrt{k_{ col }}\Vert A\Vert _2, \end{aligned}$$

which is the desired upper bound for $\Vert A\Vert _1$.

The lower bounds follow from [22, Theorem 4.2]. $\square$

The inequality (4) can also be used as a stopping criterion for iterative linear system solvers [2]. In this case, $\epsilon _\mathrm{bw}$ denotes the desired backward error, which is given prior to execution. If we replace $\Vert A-\sigma I\Vert$ with a lower bound, then we get a more stringent stopping criterion.

### Residual reduction bounds

An alternative to (4) is to use the bound

5 $$\begin{aligned} \Vert r_j\Vert \le \Vert v_j\Vert \epsilon _\mathrm{tol}. \end{aligned}$$

This bound is commonly used as a stopping condition when the linear systems are solved by iterative methods. Unfortunately, as a stopping condition, (5) “may be very stringent, and possibly unsatisfiable” [12, p. 336]. See also [9, pp. 72–73] for a $2\times 2$ example that illustrates the pitfall of comparing the norm of the residual with the norm of the right hand side. However, since (5) is de facto commonly used in computer codes it is still worth to study it under the assumption that the stopping criterion is met.

### Auxiliary residual bounds

In order to treat both (4) and  (5) in a unified way, we consider the following auxiliary bound

6 $$\begin{aligned} \Vert r_j\Vert \le \Vert v_j\Vert \epsilon _1+\Vert A-\sigma I\Vert \Vert w_j\Vert \epsilon _2. \end{aligned}$$

Clearly, the substitutions $(\epsilon _1,\epsilon _2)\leftarrow (\epsilon _\mathrm{bw},\epsilon _\mathrm{bw})$ and $(\epsilon _1,\epsilon _2)\leftarrow (\epsilon _\mathrm{tol},0)$ give back (4) and  (5), respectively. We can simplify the bound in (6) in cases when $A-\sigma I$ is not too ill-conditioned with respect to $\epsilon _2$. To see this we need the following lemma.

#### **Lemma 2**

If $\kappa (A-\sigma I)\epsilon _2<1$ and (6) hold, then

$$\begin{aligned} \Vert r_j\Vert \le \frac{\epsilon _1+\kappa (A-\sigma I)\epsilon _2}{1-\kappa (A-\sigma I)\epsilon _2}\Vert v_j\Vert . \end{aligned}$$

#### *Proof*

We have

$$\begin{aligned} \Vert r_j\Vert&\le \Vert A-\sigma I\Vert \Vert (A-\sigma I)^{-1}(v_j+r_j)\Vert \epsilon _2+\Vert v_j\Vert \epsilon _1\\&\le \kappa (A-\sigma I)\Vert v_j+r_j\Vert \epsilon _2+\Vert v_j\Vert \epsilon _1 \\&\le \kappa (A-\sigma I)(\Vert v_j\Vert +\Vert r_j\Vert )\epsilon _2+\Vert v_j\Vert \epsilon _1. \end{aligned}$$

Reordering gives the result. $\square$

The following result yields a family of new residual bounds independent of $\Vert v_j\Vert$.

#### **Proposition 3**

Let $(A-\sigma I)^{-1}(v_j+r_j)=w_j$ and assume (6) holds. If

7 $$\begin{aligned} 0 < \frac{\epsilon _1+\kappa (A-\sigma I)\epsilon _2}{1-\kappa (A-\sigma I)\epsilon _2}\le \gamma < 1, \end{aligned}$$

then

$$\begin{aligned} \Vert r_j\Vert \le \left( \epsilon _2+\frac{\epsilon _1}{1-\gamma }\right) \Vert A-\sigma I\Vert \Vert w_j\Vert . \end{aligned}$$

#### *Proof*

From (6) we have

$$\begin{aligned}\Vert r_j\Vert \le \left( \epsilon _2+\epsilon _1\frac{\Vert v_j\Vert }{\Vert A-\sigma I\Vert \Vert w_j\Vert }\right) \Vert A-\sigma I\Vert \Vert w_j\Vert .\end{aligned}$$

Thus we need to show $\Vert v_j\Vert /(\Vert A-\sigma I\Vert \Vert w_j\Vert )\le 1/(1-\gamma ).$ We have

$$\begin{aligned} \frac{\Vert v_j\Vert }{\Vert A-\sigma I\Vert \Vert w_j\Vert } = \frac{\Vert v_j\Vert }{\Vert A-\sigma I\Vert \Vert (A-\sigma I)^{-1} (v_j+r_j)\Vert } \le \frac{\Vert v_j\Vert }{\Vert v_j+r_j\Vert }, \end{aligned}$$

and from the reverse triangle inequality,

$$\begin{aligned} \frac{\Vert v_j\Vert }{\Vert v_j+r_j\Vert }\le \frac{\Vert v_j\Vert }{|\Vert v_j\Vert -\Vert r_j\Vert |}. \end{aligned}$$

Now, by Lemma 2 and assumption (7), we have

$$\begin{aligned} \Vert r_j\Vert \le \frac{\epsilon _1+\kappa (A-\sigma I)\epsilon _2}{1-\kappa (A-\sigma I)\epsilon _2}\Vert v_j\Vert \le \gamma \Vert v_j\Vert . \end{aligned}$$

Putting everything together yields

$$\begin{aligned} \frac{\Vert v_j\Vert }{\Vert A-\sigma I\Vert \Vert w_j\Vert }\le \frac{\Vert v_j\Vert }{|\Vert v_j\Vert -\Vert r_j\Vert |} \le \frac{1}{1-\gamma }. \end{aligned}$$

$$\square $$

In particular, if $\kappa (A-\sigma I)\le (1-2\epsilon _1)/(3\epsilon _2)$, then we have $\kappa (A-\sigma I)\epsilon _2<1$ and can take $\gamma =1/2$ in Proposition 3, to obtain

8 $$\begin{aligned} \Vert r_j\Vert \le (2\epsilon _1+\epsilon _2)\Vert A-\sigma I\Vert \Vert w_j\Vert . \end{aligned}$$

This is the same bound as we get from (6) if we replace $(\epsilon _1,\epsilon _2)$ with $(0,2\epsilon _1+\epsilon _2)$. In particular, if the linear systems are solved in a backward stable manner so that (4) holds, and $\kappa (A-\sigma I)\le (1-2\epsilon _\mathrm{bw})/(3\epsilon _\mathrm{bw})$, then (8) holds with $2\epsilon _1+\epsilon _2=3\epsilon _\mathrm{bw}$.

## Errors from orthonormalization

In this section we are concerned with the orthonormalization error

$$\begin{aligned} g_j=w_j-V_{j+1}h_{1:\!j+1,j}. \end{aligned}$$

Up to signs, this error can be viewed as the backward error in the $(j+1)$st column of a perturbed QR factorization

9 $$\begin{aligned}{}[v_1\; w_1\; w_2\;\cdots \;w_{k}]= V_{k+1}[e_1\;\underline{H}_k] + [0\; g_1\; g_2\;\cdots \; g_k]. \end{aligned}$$

Thus, we are interested in *columnwise* backward error bounds for QR factorizations. The next theorem shows how such bounds can be obtained from normwise backward error bounds given in the 2-norm or the Frobenius norm. It applies to floating point algorithms  that are unaffected by power-of-two column scalings, in the sense that if , then  for any $D=\text {diag}(d_1,d_2,\ldots ,d_k)$ where the $d_i$ are powers of 2. Barring underflow and overflow, this covers commonly used QR algorithms such as classical and modified Gram-Schmidt with and without (possibly partial) reorthogonalization, Householder QR and Givens QR.

### **Theorem 4**

Let  denote an algorithm that computes an approximate QR factorization of an $n\times k$ matrix *A* in floating point arithmetic. Suppose further that  for any $D= diag (d_1,d_2,\ldots ,d_k)$ where the $d_i$ are powers of 2. If *Q* and *R* denote the computed factors, $\Delta A=A-QR$ and $\Vert \Delta A\Vert _*\le \gamma \Vert A\Vert _*u,$ where $\Vert \cdot \Vert _*$ denotes the 2-norm or the Frobenius norm, then $\Vert \Delta a_i\Vert \le 2\gamma \sqrt{k}\Vert a_i\Vert u$ for $i=1:\!k$.

### *Proof*

For $i=1:\!k$, we define

$$\begin{aligned} d_i=2^{-\lfloor \log _2 \Vert a_i\Vert \rfloor }, \end{aligned}$$

so $1\le \Vert a_i\Vert d_i<2$. Since $\Delta AD$ is the backward error from  we have

$$\begin{aligned} d_i\Vert \Delta a_i\Vert&=\Vert \Delta ADe_i\Vert \le \Vert \Delta AD\Vert _*&\\&\le \gamma \Vert AD\Vert _*u <2\gamma \sqrt{k}\Vert ADe_i\Vert u = d_i2\gamma \sqrt{k}\Vert a_i\Vert u, \end{aligned}$$

for $i=1:k$, from which the theorem follows. $\square$

The constant $\gamma$ in Theorem 4 is obviously algorithm dependent and many bounds exist in the literature. Some of them contain both *n* and *k* [23], and others only *k* [1, 5], [12, Theorem 19.13]. In [12, p. 361] a columnwise bound depending on *n* and *k* is given. For Krylov methods we usually have $n\gg k$, so bounds independent from *n* should certainly be favored. We shall assume that

10 $$\begin{aligned} \Vert g_j\Vert \le \eta (n,k)\Vert w_j\Vert u, \end{aligned}$$

holds for some function $\eta (n,k)$.

### Columnwise backward errors for modified Gram–Schmidt

Our next theorem shows that for MGS, with and without one round of reorthogonalization, $\eta$ in (10) does not depend on *n* and is given by

$$\begin{aligned} \eta (n,k)=\zeta k, \end{aligned}$$

where $\zeta$ is a modest constant. We need the following forward error result for _axpy operations.

#### **Lemma 5**

Let $\alpha$ be a scalar and *x* and *y* vectors. If

$$\begin{aligned} s= \text {float} (\alpha x+y)-(\alpha x+y)\quad \text {then}\quad \Vert s\Vert \le 2(\Vert \alpha x\Vert +\Vert y\Vert )u. \end{aligned}$$

#### *Proof*

The *i*th component of $\alpha x+y$ can be viewed as the inner product $[x_i\; y_i][\alpha \; 1]^T$. Thus the componentwise forward error is bounded by $|s|\le 2u(|\alpha x|+|y|)$ [14]. We get

$$\begin{aligned} \Vert s\Vert \le \Vert 2u(|\alpha x|+|y|)\Vert \le 2 (\Vert \alpha x\Vert + \Vert y\Vert )u. \end{aligned}$$

$$\square $$

The next theorem gives columnwise backward error bounds for MGS with and without one round of reorthogonalization.

#### **Theorem 6**

Let *Q* and *R* denote the computed factors in a QR decomposition of an $n\times k$ matrix *A*, which was obtained by a floating point implementation of modified Gram-Schmidt with or without one round of reorthogonalization. Assume

(i) $$\Vert q_j\Vert =1$$ for $$j=1:\!k$$, and
(ii) $(1+(n+3)u)^{k}< 1+\delta$ for some $\delta >0$.

Then there exists a $\Delta A$ such that $A+\Delta A =QR$ with $\Vert \Delta a_j\Vert \le cj\Vert a_j\Vert u$, where $c=4(1+\delta )$ if no reorthogonalization was done and $c=10(1+\delta )^2$ if one round of reorthogonalization was done.

Let us pause for a while and discuss the assumptions before we proceed with the proof. Assumption (i) is imposed to keep our analysis cleaner; it does not affect our final bounds in any significant way. Assumption (ii) is needed for the following reason: if we compute $y=\text {float}\left( x-q_j(q_j^Hx)\right)$ for some $1\le j\le k$, then, assuming (i), the quantity $1+(n+3)u=1+\Vert q_j\Vert ^2(n+3)u$ is an upper bound for $\Vert y\Vert /\Vert x\Vert$ [12, Lemma 3.9]. Thus, (ii) guarantees that we can apply a sequence of *k* elementary “floating point” projections of the form $I-q_i^{}q_i^H$ to any vector *x*, and the resulting vector will be bounded in norm by $(1+\delta )\Vert x\Vert$.

*Proof of Theorem* 6 Let $R^{(1)}$ and $R^{(2)}$ denote the *strictly* upper triangular matrices containing the orthogonalization coefficients corresponding to the first and second round of orthogonalization, respectively. We define $R^{(2)}\equiv 0$, if no reorthogonalization is done. Assume for a while that $R^{(1)}$ and $R^{(2)}$ are given, and suppose we want to compute

$$\begin{aligned} a_j - \sum _{i=1}^{j-1} r_{ij}^{(1)}q_i - \sum _{i=1}^{j-1} r_{ij}^{(2)}q_i. \end{aligned}$$

This can be viewed as $2(j-1)$ _axpy operations. We define $a_j^{(0)} = a_j$ and

$$\begin{aligned} a_j^{(i)} = {\left\{ \begin{array}{ll} \text {float}(a_j^{(i-1)}-r_{ij}^{(1)}q_i) &{} \text {for } \quad i=1:\!j-1,\\ \text {float}(a_j^{(i-1)}-r_{(i-j+1)j}^{(2)}q_{i-j+1}) &{} \text {for } \quad i=j:\!2(j-1). \end{array}\right. } \end{aligned}$$

Using Lemma 5 yields

$$\begin{aligned} a_j^{(i)} = {\left\{ \begin{array}{ll} a_j^{(i-1)}-r_{ij}^{(1)}q_i + s_i &{} \text {for } \quad i=1:\!j-1,\\ a_j^{(i-1)}-r_{(i-j+1)j}^{(2)}q_{i-j+1} + s_i &{} \text {for } \quad i=j:\!2(j-1), \end{array}\right. } \end{aligned}$$

where

$$\begin{aligned} \Vert s_i\Vert \le {\left\{ \begin{array}{ll} 2(\Vert r_{ij}^{(1)} q_i\Vert +\Vert a_j^{(i-1)}\Vert )u &{} \text {for } \quad i=1:\!j-1,\\ 2(\Vert r_{(i-j+1)j}^{(2)} q_{i-j+1}\Vert +\Vert a_j^{(i-1)}\Vert )u &{} \text {for } \quad i=j:\!2(j-1). \end{array}\right. } \end{aligned}$$

Now, $a_j^{(i-1)}$ is also the result of applying $i-1$ elementary floating point projections to $a_j$, so the discussion prior to the proof gives $\Vert a_j^{(i-1)}\Vert < (1+(n+3)u)^{i-1}\Vert a_j\Vert$. Further, from (ii) we have $(1+nu)\Vert a_j^{(i-1)}\Vert <(1+\delta )\Vert a_j\Vert$ for $i=1:\!j-1$ and $(1+nu)\Vert a_j^{(i-1)}\Vert <(1+\delta )^2\Vert a_j\Vert$ for $i=j:\!2(j-1)$. The forward error of a computed inner product $\text {float}(x^Hy)$, where *x* and *y* are of length *n*, is bounded by $nu\Vert x\Vert \Vert y\Vert$ [14]. Thus,

$$\begin{aligned} \left| r_{ij}^{(1)} \right| \le \left| \text {float}\left( q_i^Ha_j^{(i-1)}\right) \right| \le \left| q_i^Ha_j^{(i-1)}\right| +nu \left\| a_j^{(i-1)}\right\| <(1+\delta ) \Vert a_j\Vert \end{aligned}$$

and, similarly, $\left| r_{ij}^{(2)}\right| <(1+\delta )^2 \Vert a_j\Vert$. Thus, $s_i$ is bounded by

$$\begin{aligned} \Vert s_i\Vert \le {\left\{ \begin{array}{ll} 4(1+\delta )\Vert a_j\Vert u &{} \text {for } \quad i=1:\!j-1,\\ 4(1+\delta )^{2}\Vert a_j\Vert u &{} \text {for } \quad i=j:\!2(j-1). \end{array}\right. } \end{aligned}$$

We have

$$\begin{aligned} a_j - \sum _{i=1}^{j-1} r_{ij}^{(1)}q_i - \sum _{i=1}^{j-1} r_{ij}^{(2)}q_i = a_j^{(2(j-1))}-\sum _{i=1}^{2(j-1)}s_i. \end{aligned}$$

If we define $d_i=\text {float}\left( \Vert a_j^{(2(j-1))}\Vert \right)$ and $q_j=\text {float}\left( a_j^{(2(j-1))}/d_j\right)$ and note that

$$\begin{aligned} a_j^{(2(j-1))} = q_jd_j + f_j \quad \text {with}\quad \Vert f_j\Vert \le \left\| a_j^{(2(j-1))}\right\| u<(1+\delta )^2\Vert a_j\Vert u, \end{aligned}$$

then we get

$$\begin{aligned} a_j - \sum _{i=1}^{j-1} (r_{ij}^{(1)}+r_{ij}^{(2)})q_i-d_jq_j = f_j-\sum _{i=1}^{2(j-1)}s_i. \end{aligned}$$

Finally, defining $R=\text {float(}R^{(1)}+R^{(2)})+\text {diag}(d_1,d_2, \ldots , d_k)$ yields

$$\begin{aligned} \Delta a_j := a_j - \sum _{i=1}^{j} r_{ij}q_i = f_j-\sum _{i=1}^{2(j-1)}s_i - \sum _{i=1}^{j-1} \Delta r_{ij}q_i, \end{aligned}$$

where

$$\begin{aligned} \Delta r_{ij}=r_{ij}^{(1)}+r_{ij}^{(2)} - r_{ij}, \quad \text {so}\quad |\Delta r_{ij}|\le |r_{ij}^{(1)}+r_{ij}^{(2)} |u<2(1+\delta )^{2} \Vert a_j\Vert u. \end{aligned}$$

Using the above bounds for $f_j$, the $s_i$ and the $\Delta r_{ij}$ gives $\Vert \Delta a_j\Vert < 10(1+\delta )^{2}j\Vert a_j\Vert u.$ If no reorthogonalization was done, then we have $s_i=0$ for $i=j:\!2(j-1)$, and $\Delta r_{ij}=0$, $\Vert f_j\Vert \le (1+\delta )\Vert a_j\Vert u$ for all *j*. Taking this into account yields $\Vert \Delta a_j\Vert < 4(1+\delta )j\Vert a_j\Vert u.$ $\square$

#### *Remark 1*

Suppose the perturbed QR factorization (9) was computed using MGS. Then, by taking $\delta =1/10$ and assuming that the conditions of Theorem 6 hold, we get that $\eta (n,k)$ in (10) is bounded by $\eta (n,k)\le 5 k$ if standard MGS is used, and $\eta (n,k)\le 13 k$ if MGS with one round of reorthogonalization is used. We point out that these bounds should not be interpreted as saying that standard MGS should be favored over MGS with reorthogonalization. On the contrary, as we will see in the next section, retaining a well-conditioned basis (which is the effect of reorthogonalization) is of great importance to the shift-and-invert Arnoldi algorithm.

## Backward error bounds for the shift-and-invert Arnoldi recurrence

Recall the perturbed Krylov recurrence

11 $$\begin{aligned} (A-\sigma I)^{-1}(V_k+F_k) = V_{j+1} \underline{H}_{k}, \end{aligned}$$

where $F_k = [f_1\; f_2\; \cdots \; f_k]$ and $f_j$, for $j=1:\!k$, is defined by $f_j = r_j - (A-\sigma I)g_j$. We discussed in Sects. 2 and 3 how to bound $r_j$ and $g_j$, respectively. By using these bounds, we can now easily bound $F_k$. Assuming (6) and (10) yields

12 $$\begin{aligned} \Vert f_j\Vert \le \Vert v_j\Vert \epsilon _1 + \Vert A-\sigma I\Vert \Vert w_j\Vert (\epsilon _2+\eta (n,j) u). \end{aligned}$$

Further, from (9) we see that

$$\begin{aligned} \Vert w_j\Vert = \Vert V_{j+1} h_{1:\!j+1,j} + g_j\Vert \le \Vert V_{j+1}\Vert \Vert h_{1:\!j+1,j}\Vert + \eta (n,j)\Vert w_j\Vert u, \end{aligned}$$

which in turn implies

$$\begin{aligned} \Vert w_j\Vert \le \frac{\Vert V_{j+1}\Vert \Vert h_{1:\!j+1,j}\Vert }{1-\eta (n,j)u}, \end{aligned}$$

assuming that $\eta (n,j)u<1$. We get

$$\begin{aligned} \Vert f_j\Vert \le \Vert v_j\Vert \epsilon _1 + \Vert A-\sigma I\Vert \Vert V_{j+1}\Vert \Vert h_{1:\!j+1,j}\Vert c_{jn}(\epsilon _2) \end{aligned}$$

and further (assuming that $\eta (n,k)$ is monotonically increasing in *k*)

13 $$\begin{aligned} \Vert F_k\Vert \le \sqrt{k}\Vert V_k\Vert \epsilon _1 + \sqrt{k}\Vert A-\sigma I\Vert \Vert V_{k+1}\Vert \Vert \underline{H}_k\Vert c_{kn}(\epsilon _2), \end{aligned}$$

where

14 $$\begin{aligned} c_{kn}(\epsilon _2) := \frac{\epsilon _2+\eta (n,k)u}{1- \eta (n,k)u} \end{aligned}$$

should be thought of as a tiny factor.

Similarly, if we assume the bound (8) instead of (6), we get

15 $$\begin{aligned} \Vert F_k\Vert \le \sqrt{k}\Vert A-\sigma I\Vert \Vert V_{k+1}\Vert \Vert \underline{H}_k\Vert c_{kn}(2\epsilon _1+\epsilon _2). \end{aligned}$$

This is the same bound we get from (13) if we replace $(\epsilon _1,\epsilon _2)$ by $(0,2\epsilon _1+\epsilon _2)$.

Having established (13) and (15), we are now ready to reshuffle Eq. (11) in order to derive backward error bounds for the shift-and-invert Krylov recurrence. We will derive perturbed recurrences of the form

16 $$\begin{aligned} V_k =(A+\Delta A-\sigma I)V_{k+1}\underline{H}_k. \end{aligned}$$

If we look at this from a backward error perspective, (16) means that we have taken *k* steps, without errors, of a shift-and-invert Krylov algorithm applied to a perturbed pencil, and all linear systems that occurred in the process must have been consistent. However, in order to rewrite (16) as

$$\begin{aligned} (A+\Delta A-\sigma I)^{-1}V_k =V_{k+1}\underline{H}_k, \end{aligned}$$

we need to ensure that $A+\Delta A-\sigma I$ is invertible. We need the following lemma to solve this technicality.

### **Lemma 7**

Let *A* and *V* be matrices of size $n\times n$ and $n\times k$ respectively, such that $\mathrm{rank} AV = k$. Then for any $\epsilon >0$, there exists a matrix *X* with $\Vert X\Vert <\epsilon$ such that $A+X$ is nonsingular and $XV=0$. Furthermore, if *A* is Hermitian, then we may take *X* to be Hermitian.

### *Proof*

Find a unitary matrix *Q* such that

17 $$\begin{aligned} Q^HV= \begin{bmatrix} 0 \\ V_2 \end{bmatrix} \end{aligned}$$

for some $k\times k$ matrix $V_2$, and define $AQ = [A_1\; A_2]$ where $A_2$ is of size $n\times k$. From $\mathrm{rank}AV = k$, it follows $A_2$ has rank *k*. Define *Y* so its columns span the orthogonal complement to range of $A_2$, and set $Z = [Y-A_1\; 0]$. We have that $A+ZQ^H = [Y\; A_2]Q^H$ is nonsingular and $ZQ^HV=0$. In particular, this means that the pencil $A+\lambda ZQ^H$ is regular. If $\lambda$ is any value outside the spectrum of the pencil such that $|\lambda |<\epsilon /\Vert Z\Vert$, then $X=\lambda ZQ^H$ satisfies the conditions of the theorem.

For the second part, suppose *A* is Hermitian and *Q* is such that (17) holds. Write

$$\begin{aligned} Q^HAQ=\begin{bmatrix} A_{11}^{}&\quad A_{12}^{}\\ A_{12}^H&\quad A_{22}^{} \end{bmatrix} \quad \text {and}\quad W=\begin{bmatrix} \omega I-A_{11}^{}&\quad 0\\ 0&\quad 0 \end{bmatrix}, \quad \omega >0, \end{aligned}$$

where $A_{11}$ is of size $(n-k)\times (n-k)$. We have that $QWQ^H$ is Hermitian, $QWQ^HV=0$, and

$$\begin{aligned} Q(Q^HAQ+W)Q^H = A + QWQ^H. \end{aligned}$$

Thus, for the same reason as above, it is enough to find one $\omega >0$ such that $Q^HAQ+W$ is nonsingular. Let

$$\begin{aligned} A_{22}^{}=U\begin{bmatrix} D&\quad 0 \\ 0&\quad 0 \end{bmatrix}U^H \end{aligned}$$

be a spectral decomposition where *D* is of full rank, and define $[B_1\; B_2]=A_{12}U$ such that $B_1$ has as many columns as *D*. We have that $Q^HAQ+W$ is nonsingular if and only ifis nonsingular. Further, since $[A_{12}^{T}\; A_{22}^T]^T$ is of full rank, andit follows that $B_2$ is also of full rank. We havewhich is easily seen to be nonsingular for large enough values of $\omega$. $\square$

If we use the bound on $F_k$ shown in (13), then we can deduce the following theorem.

### **Theorem 8**

Let $(A-\sigma I)^{-1}(V_k+F_k)=V_{k+1}\underline{H}_k$ be of full rank and assume $F_k$ is bounded as in (13) and $\sqrt{k}\kappa (V_k)\epsilon _1<1$. Then there is a $\Delta A$ of rank at most *k* such that

$$\begin{aligned} V_k=(A+\Delta A-\sigma I)V_{k+1}\underline{H}_k, \end{aligned}$$

and

$$\begin{aligned} \Vert \Delta A\Vert \le \sqrt{k}\Vert A-\sigma I\Vert \frac{\kappa (V_k)\epsilon _{1}+ \kappa (V_{k+1})\kappa (\underline{H}_k)c_{kn}(\epsilon _2)}{1-\sqrt{k}\kappa (V_k)\epsilon _{1}}, \end{aligned}$$

where $c_{kn}(\epsilon _2)$ is given by (14).

### *Proof*

From $V_k+F_k=(A-\sigma I)V_{k+1}\underline{H}_k$ and $V_k=(A+\Delta A-\sigma I)V_{k+1}\underline{H}_k$ we see that any eligible $\Delta A$ has to satisfy $\Delta AV_{k+1}\underline{H}_k=-F_k$. We choose $\Delta A=-F_k(V_{k+1}\underline{H}_k)^\dagger$ (which is of rank at most *k*) which implies $\Vert \Delta A\Vert \le \Vert F_k\Vert /\sigma _{ min }(V_{k+1}\underline{H}_k)$. Substituting $\Vert F_k\Vert$ by the upper bound given in (13) yields

$$\begin{aligned} \Vert \Delta A\Vert&\le \frac{\sqrt{k}\Vert V_k\Vert \epsilon _{1}+\sqrt{k}\Vert A-\sigma I\Vert \Vert V_{k+1}\Vert \Vert \underline{H}_k\Vert c_{kn}(\epsilon _2)}{\sigma _{ min }(V_{k+1}\underline{H}_k)}\\&\le \frac{\sqrt{k}\Vert V_k\Vert \epsilon _{1}}{\sigma _{ min }(V_{k+1}\underline{H}_k)}+ \sqrt{k}\Vert A-\sigma I\Vert \kappa (V_{k+1})\kappa (\underline{H}_k)c_{kn}(\epsilon _2). \end{aligned}$$

For the denominator we get

$$\begin{aligned} \sigma _{ min }(V_{k+1}\underline{H}_k)&\ge \sigma _{ min }\big ((A+\Delta A-\sigma I)V_{k+1}\underline{H}_k\big )/\Vert A+\Delta A-\sigma I\Vert \\&\ge \sigma _{ min }(V_k)/(\Vert A-\sigma I\Vert +\Vert \Delta A\Vert ), \end{aligned}$$

where we used $\sigma _{ min }(XY)\le \Vert X\Vert \sigma _{ min }(Y)$ which holds for any matrices *X*, *Y*. Thus

$$\begin{aligned}\Vert \Delta A\Vert \le \frac{\sqrt{k}\Vert V_k\Vert (\Vert A-\sigma I\Vert +\Vert \Delta A\Vert )\epsilon _{1}}{\sigma _{ min }(V_k)}+\sqrt{k}\Vert A-\sigma I\Vert \kappa (V_{k+1})\kappa (\underline{H}_k)c_{kn}(\epsilon _2) \end{aligned}$$

which can be reordered to the claimed bound. $\square$

If the linear systems are solved up to a normwise backward error $\epsilon _\mathrm{bw}$, and (8) and (15) hold for $2\epsilon _1+\epsilon _2=3\epsilon _\mathrm{bw}$, then we get the following corollary.

### **Corollary 9**

Let $(A-\sigma I)^{-1}(V_k+F_k)=V_{k+1}\underline{H}_k$ be of full rank and assume $F_k$ is bounded as in (15) with $2\epsilon _1+\epsilon _2=3\epsilon _\mathrm{bw}$. Then there is a $\Delta A$ of rank at most *k* such that

$$\begin{aligned} V_k=(A+\Delta A-\sigma I)V_{k+1}\underline{H}_k, \end{aligned}$$

and

$$\begin{aligned} \Vert \Delta A\Vert \le \sqrt{k}\Vert A-\sigma I\Vert \kappa (V_{k+1})\kappa (\underline{H}_k)c_{kn}(3\epsilon _\mathrm{bw}), \end{aligned}$$

where $c_{kn}(\cdot )$ is given by (14).

A few remarks are in order.

### *Remark 2*

If $A+\Delta A-\sigma I$ in Theorem 8 and Corollary 9 is singular, then we can invoke Lemma 7 with $V=V_{k+1}\underline{H}_k$ to obtain a backward error $\Delta \widehat{A}$, arbitrarily close to $\Delta A$, such that $(A+\Delta \widehat{A}-\sigma I)^{-1}V_k=V_{k+1}\underline{H}_k$. The new backward error $\Delta \widehat{A}$ will in general have rank greater than *k*, but its numerical rank is still bounded by *k*. Here the definition of numerical rank can be arbitrarily strict, in the sense that we may define the numerical rank as the number of singular values that greater than $\epsilon >0$, for an arbitrarily small $\epsilon$.

### *Remark 3*

If the orthonormalization is done properly, using, for instance, MGS with reorthogonalization, then $\kappa (V_{k+1})\approx 1$. In this case we can ignore the factors $\kappa (V_{k+1})$ and $\kappa (V_{k})$ when evaluating the bounds in Theorem 8 and Corollary 9. In particular this means that the bounds can be estimated cheaply as long as $\Vert A-\sigma I\Vert$ (or a good estimate of it) is known.

### *Remark 4*

For the standard eigenvalue problem, shifts are used to find interior eigenvalues, so any sensible shift satisfies $|\sigma |\le \Vert A\Vert$. Thus, we have $\Vert A-\sigma I\Vert \le 2\Vert A\Vert$ in practice.

### *Remark 5*

In view of [6], we note that our bounds do not contain the loss-of-orthonormality term $\Vert V_{k+1}^HV_{k+1}^{}-I\Vert$. Instead we saw that the condition number of the computed basis $V_{k+1}$ plays a role in the bounds of the backward error. We note, however, that a small value of $\Vert V_{k+1}^HV_{k+1}^{}-I\Vert$ implies that $V_{k+1}$ is well-conditioned:

$$\begin{aligned} \Vert V_{k+1}^HV_{k+1}^{}-I\Vert <\epsilon <1\quad \Rightarrow \quad \kappa (V_{k+1})<\sqrt{\frac{1+\epsilon }{1-\epsilon }}. \end{aligned}$$

The next example shows how Theorem 8 can be used to derive a simple a posteriori backward error bound.

### *Example 1*

Suppose a matrix *A* and a shift $\sigma$ with $|\sigma |<\Vert A\Vert$ are given, and suppose we perform *k* steps of the shift-and-invert Arnoldi algorithm. To solve the linear systems we use an iterative method that employs (5) as stopping condition, that is, the linear systems are considered “solved” when the residuals are less than some tolerance $\epsilon _{\text {tol}}$ (we ignore the norm of the right hand side since it is approximately one). We use a rather crude tolerance so $\epsilon _{\text {tol}}\gg u$. For the orthogonalization we use MGS with one round of reorthogonalization so $c_{kn}(0)\lesssim 13ku$ (cf. Remark 1). If

18 $$\begin{aligned} \epsilon _{\text {tol}}\ge \kappa (\underline{H}_k)c_{kn}(0), \end{aligned}$$

then Theorem 8, with $\epsilon _1=\epsilon _{\text {tol}}$ and $\epsilon _2=0$, and the following remarks, yield that the computed quantities satisfy

$$\begin{aligned} (A+\Delta A-\sigma I)^{-1}V_k = V_{k+1}\underline{H}_k, \end{aligned}$$

where

19 $$\begin{aligned} \Vert \Delta A\Vert \le \frac{4\sqrt{k}\kappa (V_{k+1})\epsilon _{\text {tol}}}{1-\sqrt{k}\kappa (V_k)\epsilon _{\text {tol}}}\Vert A\Vert . \end{aligned}$$

Here we have used the fact that $\kappa (V_{k+1})\ge \kappa (V_{k})$. Since MGS with reorthogonalization was employed, we expect $\kappa (V_{k+1})$ to be close to one. Thus, (19) tells us that the relative backward error $\Vert \Delta A\Vert /\Vert A\Vert$ is a modest multiple of the tolerance we used to solve the linear systems. *So, in this setting the shift-and-invert Arnoldi algorithm is backward stable.*

We end this section with a numerical experiment. We consider two matrices of order $n=1000$ and associated shifts. The first matrix is the symmetric tridiagonal matrixand the associated shift is $\sigma _1=-2$. It is well-known that the eigenvalues of $A_1$ are given by $-2+2\cos (\pi k/(n+1))$, for $k=1:\!n$, so $A_1-\sigma _1 I$ is indeed invertible. The second matrix is the nonnormal matrixalso known as the Grcar matrix [11]. The associated shift was chosen to be $\sigma _2=1$. It is an easy exercise to show that $A_2-\sigma _2 I$ is invertible.

**Fig. 1:**
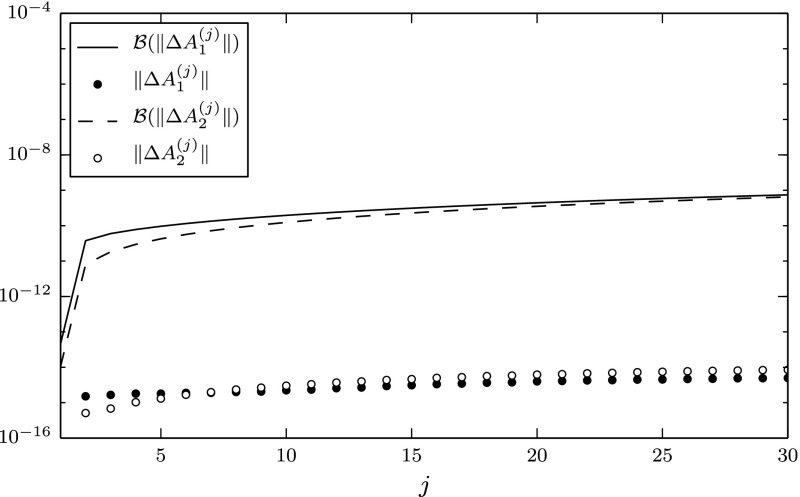
Computed backward errors and associated bound

We implemented the shift-and-invert Arnoldi algorithm in MATLABR2013a. For orthonormalization we used MGS with one round of reorthogonalization. The matrices were stored in sparse format, and the linear systems were solved using MATLAB’s “backslash” and lu routines. We took $k=30$ steps with the starting vector $[1,1,\ldots ,1]^T$, and in each iteration we computed the backward error shown in (3), where the residual was evaluated in extended precision (32 digits) and then rounded to double precision. We did this using the vpa function from the Symbolic Math Toolbox. We also computed the errors $F_k=V_k-(A_i-\sigma _i I) V_{k+1}\underline{H}_k$, $i=1,2$, in extended precision and rounded the result to double precision. For each $j=1:\!k$ and $i=1,2$, we computed

$$\begin{aligned} \mathcal {B}\left( \left\| \Delta A_i^{(j)}\right\| \right) :=\sqrt{j}\Vert A_i-\sigma _i I\Vert \kappa (\underline{H}_j)c_{jn}(3\epsilon _\mathrm{bw}), \end{aligned}$$

where $\epsilon _\mathrm{bw}$ was set to be the largest backward error of the linear systems that was encountered in the algorithm, and $c_{jn}(3\epsilon _\mathrm{bw}):=(3\epsilon _\mathrm{bw}+13ju)/(1-13ju)$ (cf. Remark 1). As is mentioned in Remark 3, the above quantity is a good estimate of the bound in Corollary 9. We also evaluated the expression for the backward errors, $\Delta A_i^{(j)} =-F_k(V_{k+1}\underline{H}_k)^\dagger$, $i=1,2$, given in the proof of Theorem 8, and computed their norms. We did this using the MATLAB routines pinv (for the Moore–Penrose pseudo-inverse) and norm. The quantities $\mathcal {B}(\Vert \Delta A_i^{(j)}\Vert )$ and $\Vert \Delta A_i^{(j)}\Vert$ are shown in Fig. 1 for $j=1:\!30$, $i=1,2$.

Our experiment seems to be unaffected by the nonnormality of $A_2$. Moreover, even though the (estimated) upper bounds $\mathcal {B}(\Vert \Delta A_i^{(j)}\Vert )$, $i=1,2$, can be seen to be rather pessimistic, they do show that the backward errors are less than $\sqrt{u}$. In other words, for both matrices, the upper bounds show that the computation is backward stable up to single precision.

## Further topics

### Implicit restarting

The bounds in Theorem 8 and Corollary 9 contain the factor $\kappa (\underline{H}_k)$, so if $\kappa (\underline{H}_k)\gg 1$ we cannot guarantee a small backward error. If we recall how Arnoldi locates eigenvalues [25, pp. 257–265], we have, unfortunately, reason to suspect that this is the case. Since Arnoldi does not target the largest eigenvalues, but *any* isolated eigenvalue cluster, $H_k:=[I_k\; 0]\underline{H}_k$ is likely to have both large and small eigenvalues, which suggests that $H_k$ may be ill-conditioned. We will now show that the situation can be much better than expected if we restrict our attention to the largest eigenvalues of $H_k$, that is, the ones corresponding to eigenvalues of *A* closest to the shift $\sigma$. The idea is to do an implicit (thick) restart [24], and purge the small eigenvalues of $H_k$. Since small eigenvalues of $H_k$ correspond to eigenvalues of *A* far from the shift $\sigma$, it is reasonable to assume they are of less interest. Suppose

$$\begin{aligned} (A-\sigma I)^{-1}(V_k+F_k)=V_{k+1}\underline{H}_k \end{aligned}$$

and consider a Schur form $H_k=QTQ^H$ such that $t_{ii}$, $i=\ell +1:\!k$, are the small eigenvalues to be purged. We have

$$\begin{aligned} (A-\sigma I)^{-1}(U_k+F_kQ)=[U_k\; v_{k+1}] \begin{bmatrix} T\\ h_{k+1,k}e_k^TQ \end{bmatrix}, \end{aligned}$$

where $U_k=V_kQ$. Throwing away the last $k-\ell$ columns yields

$$\begin{aligned} (A-\sigma I)^{-1}(U_\ell +F_kQ_\ell )=[U_\ell \; v_{k+1}] \begin{bmatrix} T_\ell \\ h_{k+1,k}e_k^TQ_\ell \end{bmatrix}, \end{aligned}$$

where $Q_\ell =Q(:\!,1:\!\ell )$, $U_\ell =U(:\!,1:\!\ell )$ and $T_\ell =T(1:\!\ell ,1:\!\ell )$. Defining $u_{\ell +1}=v_{k+1}$,

$$\begin{aligned} \underline{T}_\ell = \begin{bmatrix} T_\ell \\ h_{k+1,k}e_k^TQ_\ell \end{bmatrix}, \end{aligned}$$

and $E_\ell =F_kQ_\ell$, results in a compact recurrence

20 $$\begin{aligned} (A-\sigma I)^{-1}(U_\ell +E_\ell )=U_{\ell +1} \underline{T}_\ell , \end{aligned}$$

where $\Vert E_\ell \Vert \le \Vert F_k\Vert$. Note that our bound on $E_\ell$ depends on *k* and not $\ell$. We can now repeat the proof of Theorem 8, and use the bounds $\Vert E_\ell \Vert \le \Vert F_k\Vert$ and $\sigma _{\min }(U_{\ell +1})\ge \sigma _{\min }(V_{k+1})$, and the recurrence (20) instead of the one assumed in the theorem. We get

$$\begin{aligned} U_\ell =(A+\Delta A-\sigma I)U_{\ell +1}\underline{T}_\ell , \end{aligned}$$

where

21 $$\begin{aligned} \Vert \Delta A\Vert \le \Vert A-\sigma I\Vert \frac{\sqrt{k}\kappa (V_k)\epsilon _{1}+\sqrt{k} \kappa (V_{k+1})c_{kn}(\epsilon _2)\Vert \underline{H}_k\Vert / \sigma _{ min }(\underline{T}_\ell )}{1-\sqrt{k}\kappa (V_k)\epsilon _{1}}. \end{aligned}$$

Comparing this to the bound in Theorem 8 we see that $\kappa (\underline{H}_k)$ has been replaced by $\Vert \underline{H}_k\Vert /\sigma _{ min }(\underline{T}_\ell )$. Further, it holds that

$$\begin{aligned} \Vert \underline{H}_k\Vert /\sigma _{ min }(\underline{T}_\ell )\le \Vert \underline{H}_k\Vert /\sigma _{ min }\left( \begin{bmatrix} T\\ h_{k+1,k}e_k^TQ \end{bmatrix} \right) =\kappa (\underline{H}_k). \end{aligned}$$

It follows that if $\underline{H}_k$ is ill-conditioned due to the small eigenvalues we purged, then $\Vert \underline{H}_k\Vert /\sigma _{ min }(\underline{T}_\ell )\ll \kappa (\underline{H}_k)$ and (21) shows that the upper bound for the backward error corresponding to the part of the spectrum we care about is much smaller than the upper bound for the general backward error.

### Hermitian backward errors

We now restrict the scope to the Hermitian matrix eigenvalue problem, that is, when $A=A^H$ and $\sigma$ is real. Let us mention that we still consider the shift-and-invert Arnoldi algorithm, as it is shown in Algorithm 1, and *not* the shift-and-invert Lanczos algorithm with a three-term recurrence. In the Hermitian case, Algorithm 1 is also known as the shift-and-invert Lanczos algorithm with full orthogonalization, and it is used in, e.g., ARPACK [17, routine ssaitr.f] and MATLAB’s eigs command.

Is it, for a Hermitian *A*, possible to find a Hermitian backward error $\Delta A$? We have seen in the proof of Theorem 8 that $\Delta A$ has to satisfy $\Delta A V_{k+1}\underline{H}_k=-F_k$. Unfortunately the following lemma rules out existence of such a Hermitian $\Delta A$ in general.

#### **Lemma 10**

Let $X\in \mathbb {C}^{n\times k}$ and $F\in \mathbb {C}^{n\times k}$. Then there exists a Hermitian *E* with $E X=F$ if and only if $X^HF$ is Hermitian and $FX^\dagger X=F$. In that case, there is such an *E* with $\mathrm{rank}(E)\le 2k$ and $\Vert E\Vert _*\le 2\Vert F\Vert _*/\sigma _{\min }(X)$ where $\Vert \cdot \Vert _*$ denotes the 2-norm or the Frobenius norm.

#### *Proof*

The proof is simple and, for $k=1$, is contained in [18]. We give it for completeness. Let *E* be any matrix such that $EX=F$. This implies $EXX^\dagger X=FX^\dagger X$ and (using $XX^\dagger X=X$) $EX=FX^\dagger X$, contradicting $EX=F$ if $F\ne FX^\dagger X$. Thus $F=FX^\dagger X$ is necessary for the existence of an *E* with $EX=F$. Now, if *E* is Hermitian, then so is $X^HEX=X^HF$. Hence, if $X^HF$ is not Hermitian, then there is no Hermitian *E* with $EX=F$.

On the other hand, if $X^HF$ is Hermitian and $F=FX^\dagger X$, then

$$\begin{aligned}E:=FX^\dagger +(FX^{\dagger })^H-X^{\dagger H}F^HXX^\dagger =FX^\dagger +(FX^{\dagger })^H(I-XX^\dagger )\end{aligned}$$

is also Hermitian. Furthermore, $\mathrm{rank}(E)\le 2k$, $EX=F$, and (using that $I-XX^\dagger$ is an orthogonal projector)

$$\begin{aligned} \Vert E\Vert _*\le 2\Vert FX^\dagger \Vert _*\le 2\Vert F\Vert _*\Vert X^\dagger \Vert _2=2\Vert F\Vert _*/\sigma _{\min }(X). \end{aligned}$$

$$\square $$

The next result shows that one still gets a Hermitian backward error if one replaces the Hessenberg matrix $\underline{H}_k$ by some other $(k+1)\times k$ matrix $\underline{G}_k$. Before we state the theorem, we should clarify what we mean by “backward error” in this case. If we replace $\underline{H}_k$ by something else, we cannot say that the computed quantities ($V_{k+1}$ and $\underline{H}_k$) satisfy a an exact Krylov recurrence of a perturbed input matrix. We can, however, still say that the *computed subspace* is a Krylov subspace of a perturbed Hermitian input matrix. We refer to this Hermitian perturbation as the backward error.

#### **Theorem 11**

Let *A* be Hermitian and $(A-\sigma I)^{-1}(V_k+F_k)=V_{k+1}\underline{H}_k$. Suppose it holds for $\underline{G}_k\in \mathbb {C}^{(k+1)\times k}$ that $V_k^HV_{k+1}\underline{G}_k$ is Hermitian and $V_{k+1}\underline{G}_k$ is of full rank. Then there is a Hermitian $\Delta A$ of rank at most 2*k* such that

$$\begin{aligned} V_k=(A+\Delta A-\sigma I)V_{k+1}\underline{G}_k, \end{aligned}$$

and

$$\begin{aligned} \Vert \Delta A\Vert \le 2\frac{\Vert (A-\sigma I)\Vert \Vert V_{k+1}\Vert \Vert \underline{H}_k-\underline{G}_k\Vert +\Vert F_k\Vert }{\sigma _{ min }(V_{k+1}\underline{G}_k)}. \end{aligned}$$

#### *Proof*

From $$V_k=(A+\Delta A-\sigma I)V_{k+1}\underline{G}_k$$ and

$$\begin{aligned} V_k+F_k=(A-\sigma I)V_{k+1}\underline{H}_k=(A-\sigma I)V_{k+1}\underline{G}_k+(A-\sigma I)V_{k+1}(\underline{H}_k-\underline{G}_k) \end{aligned}$$

we see that any eligible $\Delta A$ has to satisfy

$$\begin{aligned} \Delta AV_{k+1}\underline{G}_k=(A-\sigma I)V_{k+1}(\underline{H}_k-\underline{G}_k)-F_k=V_k-(A-\sigma I)V_{k+1}\underline{G}_k. \end{aligned}$$

Since it is assumed that $V_{k+1}\underline{G}_k$ is of full rank, Lemma 10 implies that such a Hermitian $\Delta A$ exists if

$$\begin{aligned} (V_{k+1}\underline{G}_k)^H(V_k-(A-\sigma I)V_{k+1}\underline{G}_k)=(V_{k+1}\underline{G}_k)^HV_k-(V_{k+1}\underline{G}_k)^H(A- \sigma I)V_{k+1}\underline{G}_k \end{aligned}$$

is Hermitian. Since the first term on the right hand side is Hermitian by assumption, this is easily seen to be the case. Also by Lemma 10, $\Delta A$ is bounded by

$$\begin{aligned} \Vert \Delta A\Vert&\le 2\Vert (A-\sigma I)V_{k+1}(\underline{H}_k-\underline{G}_k)-F_k\Vert /\sigma _{ min }(V_{k+1}\underline{G}_k)\\&\le 2(\Vert (A-\sigma I)\Vert _2\Vert V_{k+1}\Vert \Vert \underline{H}_k-\underline{G}_k\Vert +\Vert F_k\Vert )/\sigma _{ min }(V_{k+1}\underline{G}_k), \end{aligned}$$

and is of rank at most 2*k*. $\square$

#### *Remark 6*

If $A+\Delta A-\sigma I$ is singular, then we can use the second part of Lemma 7 to find a *Hermitian* backward error $\Delta \widetilde{A}$ arbitrarily close to $\Delta A$ such that $A+\Delta \widetilde{A}-\sigma I$ is invertible.

In order to obtain a small Hermitian backward error, we need to find a matrix $\underline{G}_k$ close to $\underline{H}_k$ such that $V_k^HV_{k+1}\underline{G}_k$ is Hermitian. One possibility is

22 $$\begin{aligned} \underline{G}_k:=R_{k+1}^{-1} \begin{bmatrix} T_k\\ h_{k+1,k}e_k^T \end{bmatrix} R_k, \end{aligned}$$

where $R_k, R_{k+1}$ are upper triangular QR factors of $V_k,V_{k+1}$, respectively, and $T_k$ is the real symmetric tridiagonal matrix with $t_{j+1,j}=t_{j,j+1}=h_{j+1,j}$ and $t_{j,j}=\mathfrak {R}(h_{jj})$. Then $\underline{G}_k$ is Hessenberg and computing Ritz pairs is particularly easy: we need to find vectors *z* and scalars $\mu$ such that

$$\begin{aligned} V_k^H(A+\Delta A-\sigma I)^{-1}V_kz=\mu V_k^HV_kz. \end{aligned}$$

Here we have used Remark 6 in order to ensure that $A+\Delta A-\sigma I$ is invertible. By using the Krylov relation $(A+\Delta A-\sigma I)^{-1}V_k=V_{k+1}\underline{G}_k$ we obtain

$$\begin{aligned} V_k^HV_{k+1}\underline{G}_kz=\mu V_k^HV_kz. \end{aligned}$$

Inserting the QR factorizations $V_j=Q_jR_j$, $j=k,k+1$ and the formula for $\underline{G}_k$ shown in (22) yields

$$\begin{aligned} R_k^H[I\; 0]R_{k+1}R_{k+1}^{-1} \begin{bmatrix} T_k\\ h_{k+1,k}e_k^T \end{bmatrix}R_kz=\mu R_k^HR_kz, \end{aligned}$$

which simplifies to $T_k\tilde{z}=\mu \tilde{z}$ where $\tilde{z}=R_kz$. So, the Ritz values are just the eigenvalues of $T_k$ (which are real, since $T_k$ is Hermitian). To obtain the Ritz vectors, we would have to multiply $\tilde{z}$ with $R_k^{-1}$. However, since $R_k$ is close to the identity matrix if the orthogonalization has been done properly (for instance, by using MGS with reorthogonalization) we can approximate $\tilde{z}$ by *z*. Thus, (approximations of) Ritz pairs for the choice (22) of $\underline{G}_k$ can be obtained without computing $R_k,R_{k+1}$. We also note that choosing the eigenpairs of $T_k$ to construct Ritz pairs is what is done in practice.

### Conditions for breakdown

We now discuss how to derive a sensible breakdown criterion based on our error analysis. We saw in Sect. 1.1 that the computed quantities $V_{j+1}$ and $\underline{H}_j$ satisfy

$$\begin{aligned} (A-\sigma I)^{-1}(V_j+F_j) = V_{j+1} \underline{H}_{j}. \end{aligned}$$

This recurrence can be rewritten as

$$\begin{aligned} (A-\sigma I)^{-1}(V_j+\widetilde{F}_j) = V_{j} H_{j}, \end{aligned}$$

where $\widetilde{F}_j=F_j-(A-\sigma I)h_{j+1,j}v_{j+1}e_j^T$. Note that the first $j-1$ columns of $\widetilde{F}_j$ and $F_j$ are identical. For the last column, we have

$$\begin{aligned} \widetilde{f}_j = r_j-(A-\sigma I)(g_j + h_{j+1,j}v_{j+1}), \end{aligned}$$

where $r_j$ is the residual from the linear system and $g_j$ is associated column error from the orthonormalization. It is natural to declare breakdown when the error introduced by neglecting $h_{j+1,j}$ is of the same order as the errors that are present in the computation. This leads us to the following breakdown condition:

$$\begin{aligned} h_{j+1,j}<\Vert g_j\Vert +\Vert r_j\Vert /\Vert (A-\sigma I)v_{j+1}\Vert . \end{aligned}$$

We can simplify this condition by replacing $\Vert g_j\Vert$ with its bound in (10). This yields

23 $$\begin{aligned} h_{j+1,j}<\eta (n,j)\Vert w_j\Vert u +\Vert r_j\Vert /\Vert (A-\sigma I)v_{j+1}\Vert . \end{aligned}$$

We now discuss how to evaluate (23) in practice. If $h_{j+1,j}<\eta (n,j)\Vert w_j\Vert u$, then we can declare breakdown without further work. Otherwise we have to take the second term in (23) into consideration. If an iterative linear system solver that guarantees a residual less than some tolerance is used, then we can substitute $\Vert r_j\Vert$ in (23) by the given tolerance. If, for example, (6) is used as a stopping condition for the linear system solver, then $\Vert r_j\Vert$ is replaced by the right hand side of (6). If the residual, or any good bound for it, is not given, then we need to compute it. This is generally the case when the linear systems are solved by a direct method. Let *m* be a constant such that the following forward error bound holds for an arbitrary vector *x*

$$\begin{aligned} \Vert \text {float}((A-\sigma I)x)-(A-\sigma I)x\Vert \le m u \Vert A-\sigma I\Vert \Vert x\Vert . \end{aligned}$$

If $A-\sigma I$ is given as a dense matrix, we have $m=n^{3/2}$ [12, p. 70]. For sparse matrices, *m* can be much smaller. The computed residual $\widehat{r}_j$ satisfies

$$\begin{aligned} \Vert \widehat{r}_j\Vert&\le (1+u)\Vert \text {float}((A-\sigma I)w_j) - v_j\Vert \\&\le (1+u)(\Vert r_j\Vert +mu\Vert A-\sigma I\Vert \Vert w_j\Vert ). \end{aligned}$$

By comparing to (3), we recognize $\Vert A-\sigma I\Vert \Vert w_j\Vert mu$ as a part of the norm of a residual associated with a computed solution with corresponding backward error *mu*. Thus, we can compute a satisfactory $\widehat{r}_j$ if we use an extended precision $\overline{u}$ such that $m\overline{u}<u$.

For the computation of the vector $(A-\sigma I)v_{j+1}$, we have

$$\begin{aligned} \Vert \text {float}((A-\sigma I)v_j)-(A-\sigma I)v_j\Vert&\le m u \Vert A-\sigma I\Vert \Vert v_j\Vert \\&\le m u\kappa (A-\sigma I)\Vert (A-\sigma I)v_j\Vert , \end{aligned}$$

and, using the reverse triangle inequality, that

$$\begin{aligned} \Vert (A-\sigma I)v_j\Vert \big (1-mu\kappa (A-\sigma I)\big ) \le \Vert \text {float}((A-\sigma I)v_j)\Vert . \end{aligned}$$

Thus the norm of the computed vector is accurate enough as long as $mu\kappa (A-\sigma I)\ll 1$. If $A-\sigma I$ is so ill-conditioned that this is not satisfied, then we can use an extended precision $\overline{u}$ such that $m\overline{u}\kappa (A-\sigma I)\ll 1$.

If (6) and (23) hold, then

$$\begin{aligned} \Vert \widetilde{f}_j\Vert \le 2\big (\Vert v_j\Vert \epsilon _1+ \Vert A-\sigma I\Vert \Vert w_j\Vert (\epsilon _2+\eta (n,j)u) \big ). \end{aligned}$$

By derivations similar to those leading to (13), we get

24 $$\begin{aligned} \Vert \widetilde{F}_j\Vert \le 2\big (\sqrt{j}\Vert V_j\Vert \epsilon _1 + \sqrt{j}\Vert A-\sigma I\Vert \Vert V_{j+1}\Vert \Vert \underline{H}_j\Vert c_{jn}(\epsilon _2)\big ). \end{aligned}$$

From this we obtain the following “breakdown analogue” of Theorem 8.

#### **Theorem 12**

Let $(A-\sigma I)^{-1}(V_j+\widetilde{F}_j)=V_{j}H_j$ be of full rank and assume $\widetilde{F}_j$ is bounded as in (24) and $\sqrt{j}\kappa (V_j)\epsilon _{1}<1$. Then there is a $\Delta A$ of rank at most *j* such that

$$\begin{aligned}V_j=(A+\Delta A-\sigma I)V_{j}H_j\end{aligned}$$

and

$$\begin{aligned}\Vert \Delta A\Vert \le 2\sqrt{j}\Vert A-\sigma I\Vert \frac{\kappa (V_j)\epsilon _{1}+ \kappa (V_{j+1})\Vert \underline{H}_j\Vert /\sigma _{\min }(H_j)c_{jn}(\epsilon _2)}{1-\sqrt{j}\kappa (V_j)\epsilon _{1}},\end{aligned}$$

where $c_{jn}(\cdot )$ is given by (14).

The proof is omitted since it is essentially the same as the proof of Theorem 8. In a similar manner, we can get corresponding breakdown analogues to Corollary 9 and Theorem 11.

## Conclusion

We have shown that a floating point implementation of the shift-and-invert Arnoldi algorithm, where errors from all steps of the computation are taken into account, yields computed quantities that satisfy an exact shift-and-invert Krylov recurrence of a perturbed matrix. Here, the word “Krylov” is used instead of “Arnoldi” since the computed basis cannot be guaranteed to be perfectly orthogonal. We saw that the condition number of the computed basis $V_{k+1}$ plays a role in the bounds of the backward error. Further, we have seen that the norm of the backward error $\Delta A$ depends on $\kappa (\underline{H}_k)$. We have seen that large $\kappa (\underline{H}_k)$ are acceptable if the linear systems are only solved to a loose tolerance (18). Otherwise we argued that even if this condition number is large, the restriction to the most important part of the recurrence (that is, what is left after purging the small eigenvalues of $H_k$) can have a small backward error.

For Hermitian matrices *A*, we have shown that there is a Hermitian backward error $\Delta A$ such that the computed basis, that is, the columns of $V_{k+1}$, spans a Krylov subspace associated with $A+\Delta A$. However, as in the case of standard Arnoldi [15], the small $(k+1)\times k$ matrix associated this subspace is generally not the computed Hessenberg matrix.

Finally, we noted that our error analysis yields a sensible condition for when to declare breakdown. If this condition is met, we could derive a new set of backward error bounds, which show that an invariant subspace of a perturbed matrix has been found.
